# Olfaction and Hearing Based Mobile Robot Navigation for Odor/Sound Source Search

**DOI:** 10.3390/s110202129

**Published:** 2011-02-11

**Authors:** Kai Song, Qi Liu, Qi Wang

**Affiliations:** School of Electrical Engineering and Automation, Harbin Institute of Technology, Harbin 150001, China; E-Mails: shf67@126.com (Q.L.); wangqi@hit.edu.cn (Q.W.)

**Keywords:** multi-robot system, smell and hearing, odor tracking, sound localization, heading direction, wireless sensor networks

## Abstract

Bionic technology provides a new elicitation for mobile robot navigation since it explores the way to imitate biological senses. In the present study, the challenging problem was how to fuse different biological senses and guide distributed robots to cooperate with each other for target searching. This paper integrates smell, hearing and touch to design an odor/sound tracking multi-robot system. The olfactory robot tracks the chemical odor plume step by step through information fusion from gas sensors and airflow sensors, while two hearing robots localize the sound source by time delay estimation (TDE) and the geometrical position of microphone array. Furthermore, this paper presents a heading direction based mobile robot navigation algorithm, by which the robot can automatically and stably adjust its velocity and direction according to the deviation between the current heading direction measured by magnetoresistive sensor and the expected heading direction acquired through the odor/sound localization strategies. Simultaneously, one robot can communicate with the other robots via a wireless sensor network (WSN). Experimental results show that the olfactory robot can pinpoint the odor source within the distance of 2 m, while two hearing robots can quickly localize and track the olfactory robot in 2 min. The devised multi-robot system can achieve target search with a considerable success ratio and high stability.

## 1. Introduction

With the development of biology and computer science, bionic robots have become a hot topic in the field of intelligent robots. A bionic robot can imitate biological senses and be devoted to working with the biological modalities. Over the past few decades, the research on bionic robots has mainly focused on touch, vision and hearing. Since the 1980s, research on machine olfaction has boomed, which led to a significant advancement in biologically-inspired olfactory systems [1–4]. Bionic mobile olfactory robots are also known as active olfaction systems or active electronic noses (e-noses), which can not only perceive odors/gases such as volatile organic compounds (VOCs) [5–9], but also actively track and search for the odor/gas source. This technology shows great potential in the fields of deleterious odor source search, fire source/pollution source search, gas pipeline leak point search, combustible/explosive material detection, post-disaster search/rescue, *etc* [4,10]. In recent years, inspired by the biological olfaction, some scholars have applied e-noses and machine olfaction to mobile robots for plume tracking, odor source localization and odor distribution mapping.

Ishida imitated the way as the moth searched for the chemical bombykol, and took full advantage of odor and wind direction information to realize plume tracing [11]. Afterwards, he developed the second generation robot GaPTR-II, and proposed the transient response based algorithm [12]. Compared to the absolute gas concentration threshold based search strategy, this algorithm overcame the slow response and recovery process of metal oxide semiconductor (MOS) gas sensors. Kuwana presented a biomimetic moth pheromone tracking algorithm, namely the upwind fight method in a zigzag manner [13]. Russell used the quartz crystal microbalance (QCM) gas sensor as the antennae of a biomimetic ant in order to track a camphor odor curve smeared on the ground [14]. Subsequently, imitating the chemotaxis of *Escherichia coli* and planaria, he developed a three-dimensional space robot system, which could localize an underground odor source [15,16]. Grasso simulated the living habits of lobsters and developed an underwater robot lobster [17]. Morse imitated the chemotaxis of earthworms and used a vision sensor to perceive the luminous intensity for robot navigation [18]. Duckett employed an e-nose data trained by a neutral network to predict the distance and direction of an odor source [19]. Marques developed an e-nose composed of different types of gas sensors, which could identify a target gas from a gas mixture [20]. Lilienthal studied the method of modeling the odor distribution and made use of the gridmaps to determine the position of odor sources [21].

In recent years, not only was the gas concentration measured by gas sensors used in the search strategy, but also information from other kinds of sensors was adopted. For example, vision sensor, airflow sensor, even temperature and humidity sensors have been used in olfactory robots for target identification and obstacle avoidance [22–24]. Also, odor source searches can be accomplished by multi-robot cooperation instead of individual robots. For example, in the multi-robot system developed by Hayes *et al*., robots could measure the distribution of an odor plume and communicate with each other, and finally one of them found the odor source [25]. Ferri used a multi-robot system to search for the odor source in a repeated spiral way under weak wind conditions [26].

**Figure 1.**
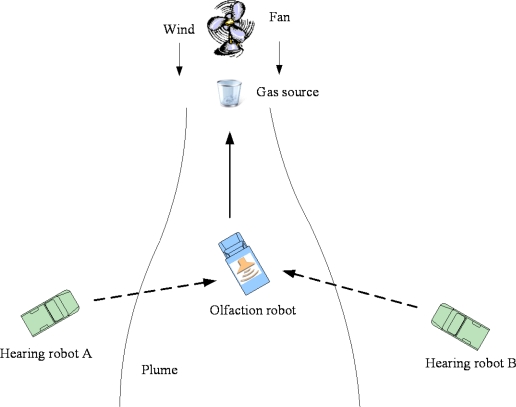
Schematic diagram of multi-robot system.

To our knowledge, research on mobile robot navigation combining smell, hearing and touch for odor/sound search has never been reported before. In this paper, we design a multi-robot system using smell, hearing and touch to track the odor/sound target. As shown in Figure 1, this system is composed of one olfactory robot and two hearing robots. Additonally, the three robots comprise a WSN and each robot is one node of the WSN. Firstly, the olfactory robot tracks the plume and searches for the odor source. Once the odor source is found, it will ring and send signals to call the other two hearing robots. Subsequently, the two hearing robots search for the sound and find their companion.

However, it is difficult to totally simulate odor/sound tracking as animal species from the perspective of bionics, since no current sensor can achieve the ability of smell and hearing of animal behaviour. For example, turbulent flow and advection always make the odor field unstable (irregular plume) so that the gas concentration detected by gas sensors fluctuates greatly [4]. Metal oxide semiconductor (MOS) gas sensor often have poor selectivity (cross-sensitivity to various odors) and strong dependence on the external environment (temperature/humidity) which will influence the accuracy of gas concentration measurements [27,28]. Besides, the response and recovery time of MOS sensors is relatively long [12], the hot-wire airflow sensor has low precision for measuring the wind direction [29], the output of sound sensor (microphone) contains environmental noise [30], and motion control of robots is imprecise [12]. Taking into account all these factors, we propose a novel heading direction based mobile robot navigation method for odor/sound tracking. The robot can adjust its heading direction according to the deviation between the current heading direction and the expected heading direction. Compared to the traditional open-loop motion control method [12], the close-loop PID motion control algorithm is used to control the robot velocity and direction continuously and steadily. The effectiveness of this method is verified by experiments. Results show that the olfaction/hearing robots can search for odor/sound source effectively and efficiently.

## 2. Olfaction Robot

### 2.1. Robot Structure

**Figure 2.**
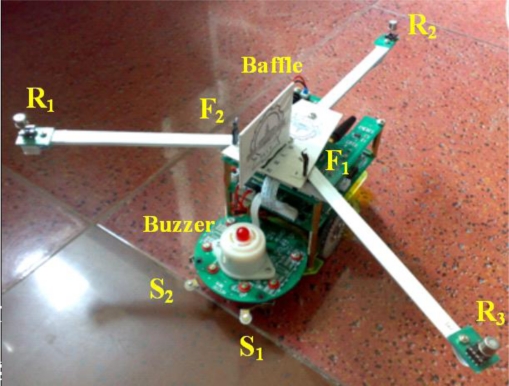
Photograph of olfactory robot.

The olfactory robot is mainly applied to track plumes and search for odor sources. Once the odor source is found, it will ring and call the two hearing robots to come. The robot is equipped with gas sensors, airflow sensors, temperature sensors, contact pickups, magnetoresistive sensor and alarm buzzer, as shown in Figure 2. Particularly, three low power consumption gas sensors R_1_, R_2_ and R_3_ (Figaro TGS2620) [31] with high sensitivity to VOCs are used for detecting gas concentration, and they are fixed on three 20 cm long extended brackets with an interval of 120°, respectively. By comparing the outputs of the three sensors, the robot can adjust its heading direction automatically. Two hot-wire airflow sensors F_1_ and F_2_ (CETC49 JFY8) serve to perceive wind velocity through the resistance changes in the wind field. They are isolated by a partition so that the robot can keep moving upwind by balancing the wind velocities of its left and right sides. In order to make temperature compensation for each anemometer, a compensation bridge circuit is designed using Pt resistor temperature sensor Pt1000. Besides, an integrated-circuit semiconductor temperature sensor (National Semiconductor LM35) is used to measure the ambient temperature. Two contact pickups S_1_ and S_2_ comprising microswitch, electric relay and transistor are employed to perceive whether the robot collides with the odor source. When the robot hits against the odor source, the alarm buzzer will be triggered instantly. The magnetoresistive sensor (Honeywell HMC1022) is used to calculate the robot heading angle by measuring the horizontal and vertical two-axis magnetic field strength [32].

The robot is assembled with 150 MHz TI TMS320F28335 [33], a high-speed 32 bits floating point digital signal processor (DSP), which integrates a 12-channel and 12-bit A/D converter and 18 pulse width modulation (PWM) units [28]. Compared with the previous generation TI DSP, the performance is improved by approximately 50%. As the core of the robot, the DSP processor acquires all sensor outputs, performs signal processing, executes multi-sensor fusion search algorithm, drives the motor, adjusts velocity and course of the robot and controls it to search for the odor source step by step. Besides, the DSP controller communicates with the wireless communication unit (WCU) via a serial communication interface (SCI).

The WCU of olfaction robot realizes information transmission with the sink node connected to PC and the two hearing robots through a wireless RF transceiver. Its major tasks include three parts: (1) receive the control command from PC to start the odor tracking task (2) transmit course information (current heading angle as well as expected heading angle) and ambient information (gas concentration, wind direction, temperature, *etc.*) to the PC for displaying the angular position of the robot graphically (3) inform the two hearing robots to start their sound localization tasks when the olfactory robot finds the odor source. A low power consumption ZigBee single chip (TI CC2430) [28] is selected as the wireless communication transceiver, which integrates 2.4 GHz RF transceiver and digital received signal strength indicator (RSSI) [34]. RSSI can measure the distance between arbitrary two robots (WSN nodes) through the received signal intensity.

The structure of the robot is based on a three-wheeled vehicle which is mainly composed of two driving wheels, an all-direction wheel and two motors that are connected to the two driving wheels respectively. Rotation direction of each motor is controlled by the direction of drive current and rotation velocity is controlled by the duty cycle of PWM. Thanks to the two driving motors and the above structure, the robot can move forwards and turn left or right simply and flexibly [35]. Since electromagnetic interference seriously affects the accuracy of sensor data acquisition during the motor run, an independent power supply is designed for motor driving. The motor control signals are isolated through the optoelectronic isolators so that electromagnetic interference is reduced.

### 2.2. Distribution of Odor Field

Before the olfaction robot tracks the plume, it is important and instructive to build the distribution model of the odor field. Since molecule diffusion velocity is always slower than wind velocity *u*, the plume is mainly determined by air advection in the case of large winds (e.g., *u* > 1.5 m/s). Thus the concentration distribution equation can be established using the Gaussian diffusion model [20]. In the case of stable breeze (e.g., *u* < 1.5 m/s), the plume is primarily affected by air turbulence and can be estimated using turbulent diffusion equation [36]. As the wind velocity decreases, the turbulence is weakened too. When the wind velocity decreases to 0 m/s, the plume structure is only ascertained by molecular diffusion [15]. This paper deals with the indoor plume distribution under the stable breeze condition (1.5 m/s > *u* > 0.5 m/s), and builds the gas concentration distribution equation based on the integral model of mobile puff diffusion [37]:

(1) $$\begin{array}{ll} C\left(x,y,z,H\right)= & \int_{0}^{+\infty }\frac{Q}{{\left(2\pi \right)}^{\frac{3}{2}}\sigma_{x}\sigma_{y}\sigma_{z}}\cdot \text{exp}\;\left[-\frac{{\left(x-\mathrm{ut}\right)}^{2}}{2\sigma_{x}^{2}}\right]\cdot \;\text{exp}\;\left(-\frac{y^{2}}{2\sigma_{y}^{2}}\right)\cdot \\ & \left\{\text{exp}\;\left[-\frac{{\left(z-H\right)}^{2}}{2\sigma_{z}^{2}}\right]+\text{exp}\;\left[-\frac{{\left(z+H\right)}^{2}}{2\sigma_{z}^{2}}\right]\right\}\mathrm{dt} \end{array}$$

where *C* is the gas concentration, *Q* is the gas source strength, *u* is the average wind velocity, *x* is the downwind distance, *y* is the crosswind distance, *z* is the gas source distance from the ground, *H* is the effective gas source height, *σ_x_*, *σ_y_* and *σ_z_* are the standard deviations of gas concentration and denote the diffusion parameters of direction *x*, *y* and *z* respectively and *t* is the puff movement time.

Note that Equation (1) represents the gas concentration of a point in downwind space under the bounded conditions. The ground projection coordinate of odor source is origin (0,0), and positive *x*-axis points to the wind direction. The three parameters *σ_x_*, *σ_y_* and *σ_z_* are expressed as follows:

(2) $$\begin{array}{l} \sigma_{x}=\sigma_{y}=\gamma_{1}t\\ \sigma_{z}=\gamma_{2}t \end{array}$$

where constant *γ*_1_ and *γ*_2_ represent regression coefficients of the diffusion parameters in the horizontal and vertical directions respectively and can be obtained from the data table. *η*^2^ and *s* are defined below:

(3) $$\eta^{2}=x^{2}+y^{2}+\gamma_{1}^{2}H^{2}/\gamma_{2}^{2}$$

(4) $$s=\frac{\mathrm{xu}}{\eta \gamma_{1}^{2}}$$

When *H* = 0, substitute Equations (2–4) into Equation (1) and introduce the normal distribution function:

(5) $$\Phi \left(s\right)=\frac{1}{\sqrt{2\pi }}\int_{-\infty }^{s}\;\text{exp}\;\left(-\frac{t^{2}}{2}\right)\mathrm{dt}$$

Thus the diffusion model of ground continuous point-source can be described as:

(6) $$C\left(x,y\right)=\frac{2Q}{{\left(2\pi \right)}^{\frac{3}{2}}\gamma_{2}\eta^{2}}\;\text{exp}\;\left(-\frac{u^{2}}{2\gamma_{1}^{2}}\right)\cdot \left[1+\sqrt{2\pi }\;\cdot \;s\;\cdot \;\text{exp}\;\left(\frac{s^{2}}{2}\right)\cdot \Phi \left(s\right)\right]$$

where Φ(*s*) can be solved through numerical integration or look-up table.

**Figure 3.**
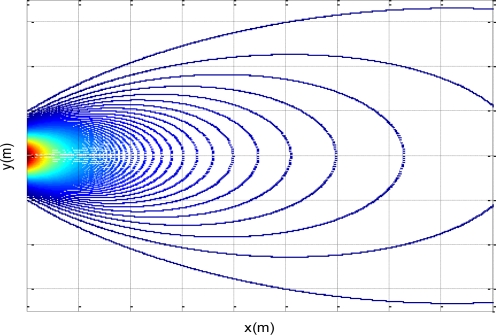
Concentration gradient curves of plume model.

According to Equation (6), if the coordination (*x*,*y*) of a point in the plume is known, the corresponding gas concentration of that point can be calculated. The concentration gradient curves drawn in Matlab software are shown in Figure 3. Actually, the plume shape is irregular and concentration distribution of odor field is disturbed by the turbulent flow in a real environment.

### 2.3. Odor Source Tracing

In the previous section, we discuss the plume model under different conditions (e.g., calm/breeze/gale) and particularly deduce the integral model of mobile puff diffusion in the breeze condition. In this section, the odor source tracing methods are presented. Firstly, plume tracing strategies in different circumstances are preliminarily designed according to the different wind velocities. Secondly, the principle of heading direction based robot motion control is introduced. The olfaction robot uses magnetoresistive sensor, PID controller and PWM mapping to execute a closed-loop control for its velocity and direction. Finally, the multi-sensor fusion step-by step search algorithm as the real tracking strategy of the olfactory robot is described in detail.

#### 2.3.1. Preliminary Design of Search Strategy

As mentioned above, the odor plume is dispersed into a round field in the calm condition, and concentration gradient curves approximate a series of concentric circles. Since the middle concentration is much higher than the edge concentration, the robot can find the odor source according to the concentration gradient [15]. In the condition of high wind velocity, as the influence of airflow is greater than molecular diffusion, the odor distribution is mainly determined by wind and the plume is relatively narrow. The robot can track the odor source in a zigzag pattern by judging the edge of the plume [4,12]. In the case of low wind velocity, as the odor distribution is led by air turbulence and molecular diffusion together, the plume is relatively wide in the crosswind direction [37]. Based on the information fusion from gas sensors and airflow sensors, the robot can search for the odor source upwind or along the gas concentration gradient adaptively. During the search process, these two strategies are converted from one to the other. However, note that the strategy under the breeze condition needs two prerequisites. Firstly, the movement direction/course of the robot should be measured and controlled accurately so that the robot is able to move towards the target. Secondly, the thresholds of airflow sensors and gas sensors used in the strategy conversion should be properly set, and the tracking process is better to be divided into several phases in order to improve the search success ratio.

#### 2.3.2. Heading Direction Based Motion Control

When the robot moves at a high speed in the odor plume, the slow response and recovery of gas sensors lower the accuracy of gas concentration measurement and have negative effects on search success ratio, whereas slowing down the robot will increase the search time. Therefore, it is crucial to accurately control velocity and direction of the robot. In this paper, we describe a heading direction based motion control method to navigate the robot [35,38]. The robot can automatically and stably adjust its speed and direction according to the deviation between the current heading direction and the expected heading direction. The computation flow of this method is described as follows:

##### (1) Computation of Current Heading Angle

Supposing the robot is placed on the horizontal plane namely (*x*,*y*) plane, the current heading angle can be calculated via the north magnetic pole. A two-axis intelligent weak magnetic chip (Honeywell HMC1022) [32] is used to measure the magnetic components *H_x_* and *H_y_* (neglecting the robot’s incline in *z*-axis). HMC1022 including two mutually perpendicular magnetoresistive sensors in its internal is assembled on the circuit with its *x*-axis consistence with the heading direction of the robot. Here, *H_x_* and *H_y_* are acquired by A/D converter of DSP, and the current heading angle *α* is computed by:

(7) $$\alpha ={\text{tan}}^{-1}\left(\frac{H_{y}}{H_{x}}\right)$$

##### (2) Computation of Expected Heading Angle

The olfactory robot determines its next steering angle *θ* by fusing the information of wind velocity and gas concentration. Here, the expected heading angle *β* is defined as the sum of steering angle *θ* and current heading angle *α*. The movement velocity and heading direction of the robot can be continuously and smoothly changed by the PID control algorithm in order to make the updated current heading angle close to the expected heading angle precisely.

##### (3) Robot Motion Control

The position PID control algorithm serves to the robot navigation. Here, *β* is obtained in the previous step, and *α* is calculated once per 10 ms. After that, the heading angle deviation *γ* can be computed by subtracting the updated *α* from *β*, then the PID control algorithm is executed instantly. The output *ɛ* of PID controller is computed using the program in Table 1. According to the robot structure and the motor property, PID parameters K_P_, K_I_, K_D_ are set at 2, 0.5 and 0.01 respectively, and the output *ɛ* is limited to ±100°.

A TI L293D chip is used as the driver to drive the two motors of the robot. The DSP controller is capable of adjusting motor velocity by changing the PWM duty cycle input into the L293D and employing general I/O pins to alter motor rotation direction. It is necessary to build a mapping between PID output *ɛ* and PWM duty cycle (Table 2). The error threshold *L* is set at 50 according to the motor performance, and the robot velocity is controlled at 10–20 cm/s under the advisable duty cycle. When | *ɛ* |> *L*, since error between the current heading direction and the expected heading direction is large, two motors run in the opposite direction in order to make the robot rotate quickly. Conversely, when | *ɛ* |< *L*, since error between the current heading direction and the expected heading direction is small, two motors run in the same direction in order to make the robot move forward. Table 3 shows the robot motion states.

#### 2.3.3. Multi-Sensor Fusion Step-by-Step Search

Generally, it is complex for a robot to search for the odor source, since molecular diffusion is slower than air flow, and the odor plume always forms downwind from the odor source. With the plume extension, massive vortexes and twists appear, which make the gas concentration unstable [4]. Therefore, a search algorithm based only on the gas concentration gradient ceases to be effective under such circumstances. In this paper, the devised olfactory robot achieves plume tracing step by step using multi-sensor information including gas concentration, wind velocity, temperature, heading angle, *etc*. The basic program flow is as follows. Firstly, the robot judges whether it is in the odor field through gas concentration measured by gas sensors. Secondly, if the robot is in the odor field, it computes the wind direction via the wind velocity detected by airflow sensors and searches for the odor source upwind or determines the maximal concentration direction according to the concentration gradient. Finally, the robot tracks the plume via heading direction in combination with PID control algorithm which can control its velocity and direction accurately.

Figaro TGS2620 is a class of MOS gas sensor based upon resistance changes and has high sensitivity to organic solvents or other volatile odors [31]. The sensor output is defined as resistance ratio *R_s_*/*R_0_*, where *R_s_* is the sensor resistance in the test gas, *R_0_* is the sensor resistance in air. The definition is to weaken environmental impact on sensor response and improve the accuracy of concentration measurement [39,40]. When the gas concentration increases, the resistance ratio decreases. Three gas sensors form the ‘Y’ shape with an interval of 120° and are fixed on three 20 cm long extended brackets respectively, as shown in Figure 2. R_3_ and R_1_ installed in the front are used to control the robot to turn left or right, and R_2_ in the back is used to reverse the course when the robot runs away from the odor field. Thus the robot can measure gas concentrations of three different positions simultaneously in order to compute the concentration gradient. Compared to the layout in [12] (three gas sensors are fixed on one horizontal beam), this ‘Y’ shape can not only extend measurement space for improving the precision of concentration gradient but also shorten the search path for enhancing the odor tracking efficiency.

As mentioned above, the response and recovery of MOS gas sensors are relatively slow [12]. For example, once the robot gets into the odor field and makes contact with the gas, the output of gas sensor reduces, and it needs about 5–10 s to reach a stable state. Homogeneously, when the robot runs away from the odor field, the gas sensor output increases, and it takes approximately 15–20 s to be stable [28]. Therefore, the robot cannot move continuously during the search process, it has to stop and wait about 15 s to acquire odor information for processing and comparing, and then decide the next steering angle according to the multi-sensor information fusion results. This kind of movement pattern guarantees a high success ratio for the search task but it expends much search time. In addition, although the plume distribution is relatively stable in a constant wind field, other factors such as indoor air turbulence, convection and man-made walking interference will result in the plume fluctuation and make it more difficult for the robot to search for the odor source. For these reasons, trimming extreme values and smoothing filter technology is used in this algorithm.

A constant current hot-wire airflow sensor is used to measure the wind velocity through the resistance variation. Since air flow takes heat away in the wind field, airflow sensor resistance decreases as working temperature declines when it is heated to a high temperature by constant current [29]. In order to eliminate the ambient effects and improve the measurement accuracy, a compensation bridge circuit composed of Pt resistance Pt1000 and potentiometer is designed for temperature compensation. When the environment temperature varies, both of the airflow sensor resistance and the Pt resistance change so that temperature is compensated. Because it is unnecessary to acquire the absolute wind velocity which needs to be calibrated precisely in the wind tunnel, the robot can measure the relatively wind velocity indirectly by taking the voltage of differential amplification circuit as the output of the airflow sensor. As shown in Figure 2, two airflow sensors F_1_ and F_2_ are assembled symmetrically at both sides of the symmetry axis of the robot and forms a 90° included angle with the robot’s geometry central point. F_1_ is on the left side, F_2_ is on the right side, and they are isolated from the middle by a partition in order to determine the wind direction. The larger the voltage value of which side the higher is the wind velocity. Thus the wind direction can be determined.

**Figure 4.**
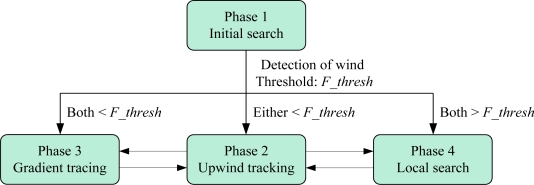
Block diagram of multi-sensor fusion step-by step search algorithm.

The block diagram of the multi-sensor fusion step-by-step search algorithm is shown in Figure 4. The entire process is divided into four phases. Phase 1 is the initial search. During this phase, the olfaction robot detects a gas concentration via three gas sensors and judges whether it is in the odor plume according to the initial threshold *R_init*. It is manifested that the robot itself is in the plume if all outputs of the three gas sensors are less than *R_init*. Otherwise, the robot will continue to track the plume and move towards the sensor direction in which the gas concentration is highest. In phase 2, the robot searches for the odor source upwind since the gas concentration increases along the upwind direction. The threshold *F_thresh* is set for the two airflow sensors. If one is above *F_thresh*, and the other is below *F_thresh*, the robot will move towards the direction where the output is higher. If both of the velocities measured are below *F_thresh*, the process turns to phase 3. In phase 3, the robot searches for the plume based on gas concentration gradient. The threshold *R_turning* is set for the outputs of gas sensors R_1_ and R_3_ and is used to help the robot determine the next steering angle. If the minimum of R_1_ and R_3_ is less than *R_turning*, the robot will move towards the direction of the sensor that outputs the minimum. Otherwise, the robot will continue to move forward in a straight line. Occasionally, it is possible for the robot to move to a wrong direction so that it may run away from the plume. In that case, sensor R_2_ plays its role as a redirector. The threshold *R_ reverse* is set for the gas sensor R_2_. If the output of R_2_ is the minimum among the three gas sensors and is below *R_ reverse*, the robot will turn back to the plume again. In the end of upwind tracking, as the robot is close to the wind source, both of the outputs of F_1_ and F_2_ may be above *F_thresh*, by this time, the process turns to phase 4—local precise search. The search strategy is similar to that of phase 3. By comparing the outputs of R_1_ and R_3_, the robot can steer at a small angle or move forward towards the odor source. If the outputs of airflow sensors satisfy the threshold condition of phase 2 during phase 3 or phase 4, the robot will search upwind again, that is, phase 2, phase 3 and phase 4 are interconvertible as shown in Figure 4.

In summary, the robot can obtain the current heading angle via magnetoresistive sensor and acquire the expected heading angle by information fusion from three gas sensors and two airflow sensors. According to the deviation between the current heading angle and the expected heading angle, the robot can finally adjusts its speed and course by direction angle error of PID output and PWM duty mapping.

## 3. Hearing Robot

### 3.1. Robot Structure

Once the olfactory robot finds the odor source, the buzzer is triggered to alarm by the electric microswitch. Simultaneously, the robot sends the startup commands to two hearing robots via WSN and informs them to come. Each hearing robot is equipped with microphone array for sound gathering and localization. As one node of WSN, when the individual hearing robot receives the action command, it will start the sound localization/search program, and then gradually move towards the olfaction robot.

**Figure 5.**
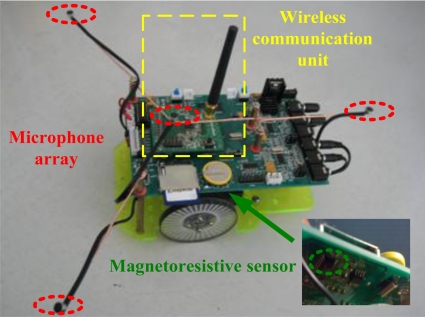
Photograph of hearing robot.

A photograph of a hearing robot is shown in Figure 5. Each hearing robot is composed of three parts: microphone array and audio processing circuits, DSP signal processing unit as well as WCU. Four microphones are installed as a ‘Y’ shape on three 15 cm long brackets respectively with an interval of 120°. Such an arrangement is beneficial for sound localization [35]. Taking the intermediate microphone as the center, the robot can estimate the position and direction of sound source by computing the time delay between arbitrary two microphones. As the core of hearing robot, the DSP processor performs signal acquisition and processing, sound localization and search, motor drive and control, serial communication with WCU, *etc*. WCU realizes information transmission with the olfactory robot and the sink node connected to PC. Its major tasks include: (1) receive startup command from the olfactory robot and start the sound localization/search program for tracking the olfactory robot (2) transmit robot heading angle, distance from the sound source, sound source direction angle and search result to PC (3) measure the distance between two robots via RSSI and avoid their collision.

According to the large-scale path loss model, the relation between received signal strength *R* and transmission distance can be described as [34]:

(8) $$R=R_{0}-10r\;\text{log}\;\left(\frac{d}{d_{0}}\right)+\xi$$

where *R*_0_ is the signal strength in the distance *d*_0_ from the emitting node, *d* is the distance between the emitting node and the received node, *r* is a parameter related to signal propagation environment and is generally in the range of 2–4, and *ξ* is the random noise with mean 0 and standard deviation 4–10. Here, a threshold *D_thresh* is set. If the distance *d* between two nodes (robots) is less than *D_thresh*, the hearing robot will stop its search in order to avoid collisions.

### 3.2. Sound Localization and Search

Sound localization and search algorithms include three parts—TDE of microphone array, spherical interpolation based sound localization as well as sound source search algorithm using course information. Firstly, TDE between arbitrary two microphones is computed through generalized cross correlation (GCC) [41,42]. Secondly, sound location is determined by spherical interpolation [43] in combination with the geometrical position of microphone array. Finally, sound direction angle is computed and the sound search mission is accomplished by PID control algorithm. The principle and program flowchart of the three algorithms are described below.

#### 3.2.1. Time Delay Estimation of Microphone Array

Assume that *D* is the distance between two microphones M_1_ and M_2_. Their received signals *x*_1_(*n*) and *x*_2_(*n*) from the same sound source without reverberation can be written as:

(9) $$x_{1}(n)=a_{1}s(n-\tau_{1})+w_{1}(n)$$

(10) $$x_{2}(n)=a_{2}s(n-\tau_{2})+w_{2}(n)$$

where *s*(*n*) is the sound source signal, *τ*_1_, *τ*_2_ is the acoustic transmit time from sound source to two microphones, *a*_1_, *a*_2_ are the acoustic attenuation coefficients, and *w*_1_(*n*), *w*_2_(*n*) are the irrelevant Gaussian white noises. The cross correlation function *R*_12_ (*τ*) of *x*_1_(*n*) and *x*_2_(*n*) can be expressed as:

(11) $$R_{12}(\tau )=E(x_{1}(n)x_{2}(n-\tau ))$$

where *E* denotes mathematic expectation.

Define *τ*_12_ = *τ*_1_ – *τ*_2_ as the time delay of two microphones. As *w*_1_(*n*) and *w*_2_(*n*) are irrelevant Gaussian white noises, *s*(*n*) and *w*(*n*) are irrelevant stochastic signals. Then *R*_12_ (*τ*) can be transformed by substituting Equations (9) and (10) into Equation (11):

(12) $$R_{12}(\tau )=E(a_{1}a_{2}s(n-\tau_{1})s(n-\tau_{2}-\tau ))=a_{1}a_{2}R_{s}(\tau -\tau_{12})$$

where *R_s_* (*τ* – *τ*_12_) is the autocorrelation function of *s*(*n*). According to the property of correlation function, *R*_12_ (*τ*) has the maximum when *τ* = *τ*_12_. By the relation between cross correlation function and cross power spectrum, *R*_12_ (*τ*) can be described as:

(13) $$R_{12}(\tau )=\int_{0}^{\pi }G_{12}(\omega )e^{-j\omega \tau }d\omega$$

where *G*_12_ (*ω*) is the cross power spectrum of *x*_1_(*n*) and *x*_2_(*n*). Therefore, *R*_12_ (*τ*) can be computed by the inverse transform of *G*_12_ (*ω*).

**Figure 6.**
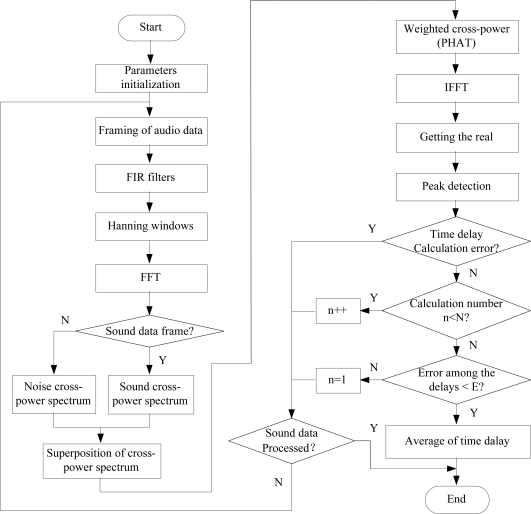
Flowchart of TDE.

However, since the peak value of *R*_12_ (*τ*) is affected by noise so that it is not obvious as expected, and the estimation accuracy of *τ*_12_ is largely reduced. To sharpen the peak value of *R*_12_ (*τ*), it is effective to weight *G*_12_ (*ω*) in frequency domain. The phase transformation weighted function is employed in this paper for reducing the effects of noise and reverberation. Cross correlation function can be obtained via inverse fast Fournier transformation (IFFT). Then *τ*_12_ can be obtained by disposing the real part of cross correlation function based on peak detection method. Since there are four microphones in each hearing robot, we can get a pair of time delay by picking any two microphones so that there are totally $C_{4}^{2}=6$ pairs of time delays. The detailed program flowchart of TDE is shown in Figure 6.

#### 3.2.2. Spherical Interpolation Based Sound Localization

**Figure 7.**
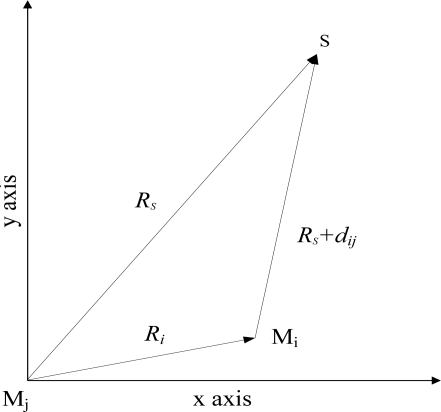
Geometry relations among M_i_, M_j_ and S.

In the preceding section, TDE of microphone array has been acquired using GCC method. This section elaborates the method of sound localization. Here, spherical interpolation as the method of sound localization is used to solve a set of equations based on TDE and geometric relation of microphone array under the least-squares formula [35,43]. The detailed process of this method is described as follows: we first map the spatial origin into arbitrary two microphones M_i_ and M_j_, whose geometrical relations with sound source S are shown in Figure 7, where ***r****_i_* is defined as the vector from M_j_ to M_i_, and the distance *R_i_* between two microphones is denoted *R_i_* =| ***r****_i_* |. Analogously, ***r****_s_* is the vector form M_j_ to S, and *R_s_* =| ***r****_s_* | is the distance between M_j_ and S. Then define *d_ij_* as the distance difference from S to M_i_ and M_j_. Therefore, Equation (14) can be set up using vector geometry and triangle trilateral relations:

(14) $${\left(R_{s}+d_{\mathrm{ij}}\right)}^{2}=R_{i}{}^{2}-2r_{i}{}^{T}r_{s}+R_{s}{}^{2}$$

and:

(15) $$0=R_{i}{}^{2}-d_{\mathrm{ij}}{}^{2}-2R_{s}d_{\mathrm{ij}}-2r_{i}{}^{T}r_{s}$$

As TDE is typically not measured precisely and *d_ij_* is computed by TDE, Equation (15) does not equal zero at its left side. Then it becomes:

(16) $$ɛ=R_{i}{}^{2}-d_{\mathrm{ij}}{}^{2}-2R_{s}d_{\mathrm{ij}}-2r_{i}{}^{T}r_{s}$$

Assume that the number of microphones is *m*, which is denoted 0,1,…,*m* − 1. Therefore, the distance difference from microphone 0 to other microphones designated by 1,2,…,*m* − 1 can be expressed. Also, the corresponding *m* − 1 sets of equations can be set up. When the mean squared error has the minimum, *r_s_* is the best estimation of sound source position [35].

#### 3.2.3. Sound Source Search

According to the estimation of sound source position, the hearing robot can compute the deflection angle of sound source referenced to its current heading angle, and the deflection angle *θ* is defined as the sound source direction angle. Then the expected heading angle *β* is set as the sum of the current heading angle *α* which is measured by the magnetoresistive sensor, and the sound source direction angle. The control principle of the hearing robot is similar to that of the olfactory robot, described in Section 2.3.2. By operating the heading angle deviation *γ* using PID control algorithm, the direction angle error *ɛ* (*i.e*., the output of PID controller) can be acquired, then the velocity and heading direction of the hearing robot can be controlled through the PWM duty cycle mapping.

**Figure 8.**
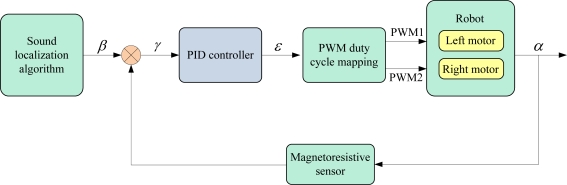
Closed-loop control diagram of hearing robot for sound search.

This closed-loop control diagram is shown in Figure 8. Since *α* is updated repeatedly and duty cycle is computed continuously, robot motion is adjusted gradually and steadily. Particularly, when the direction angle error is large, duty rates of the two motors are large, and two motors are operated in the opposite direction in order to make a quick rotation. When the direction angle error is small, duty rates of the two motors are set small, and two motors are operated in the same direction so as to realize an angle fine-tuning. Therefore, the hearing robot can find the sound source (olfactory robot) by executing the search algorithm repetitively.

### 3.3. Nodes Communication in WSN

As previously mentioned, both the olfactory robot and the two hearing robots are equipped with WCU, and data communication between WCU and DSP are achieved via SCI [28]. Thanks to the RF transceiver and RSSI, each robot can exchange information and measure the distances from the other two robots. Three robots as the slave nodes and the sink node connected to PC together constitute a WSN. In order to transport information effectively and reliably in the WSN, the whole system adopts a uniform data communication protocol, in which, each data frame contains nine bytes. As shown in Table 4, FA and FB is the frame head and Addr is the address bit. Addr is 1 for olfactory robot, 2 for hearing robot 1, 3 for hearing robot 2, respectively, and is 4 for the sink node. Fn is the function bit that represents the implication of the transmitted data (Table 5). Bit1, Bit2, Bit3, Bit4 are the valid data bits that represent the specific values of the transmitted data. Check is the frame end and is the sum-check bit too. Data frame should be discarded if the checksum is invalid.

## 4. Experimental Results and Discussion

### 4.1. Experimental Environment

**Figure 9.**
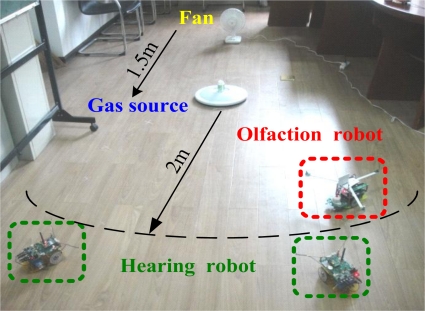
Photograph of the experimental environment.

Odor/sound source search were conducted in a clear and quiet room. In order to minimize the ambient airflow impact on the experiments, it was necessary to close doors and windows, and reduce man-made interference during the experiments. In addition, to avoid noise effects on sound localization, air conditioner and other ventilator were turned off. The experiment setup is shown in Figure 9.

The experiment arena spanned 5 (length) × 3 (width) m^2^ and was surrounded by walls and desks. A small fan was laid to create a stable wind field, and a small bottle of 93# gasoline used as the odor source was placed 1.5 m downwind from the fan. The wind velocity was controlled in the range of 0.5–1.5 m/s (*i.e*., breeze condition) so that a steady odor field could easily are formed. The olfactory robot and two hearing robots were 2 m downstream from the odor source.

Running states and information of the three robots acquired from sensors were continuously sent to PC via WSN and were visible on the PC software interface for monitoring. Also, robots communicated with each other and cooperated to accomplish the odor/sound search task. The entire process of experiments is summarized as follows.

The olfactory robot searched for the odor source firstly. Once it found the target, it alarmed and informed the two hearing robots to come. Then the two hearing robots localized and searched for the sound source (*i.e*., olfactory robot). The experimental results of odor/sound tracking are described in the following sections.

### 4.2. Odor Source Tracking Trial

**Figure 10.**
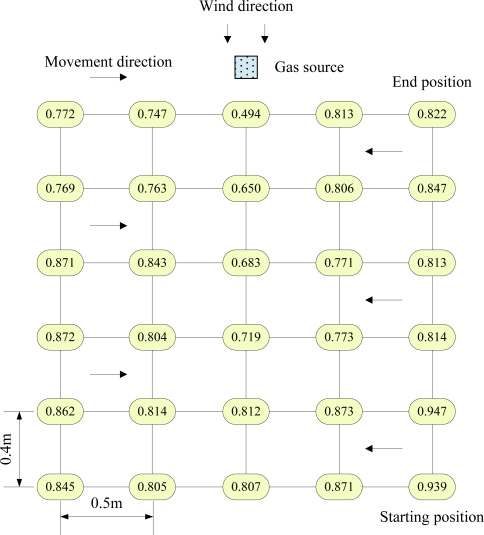
Odor concentration distribution in the experimental arena.

Before an odor source search, it is essential to study the plume distribution for determining the parameters of the search algorithm. Therefore, the olfactory robot was employed to collect the gas concentration at each specific point of odor field in advance. A gas concentration profile was drawn according to the acquired data. The details of concentration acquisition are presented as follows: the olfactory robot was first set in the manual mode. Then it moved to each assigned sample point to acquire the gas concentration and transmitted the data to the PC via the WSN. Note that the response *R_s2_*/*R_02_* of gas sensor 2 was defined as gas concentration. The robot movement path and odor plume distribution are shown in Figure 10. Measurement value of each sample point was averaged over 30 s. There were 30 sample points in the 2 m × 2 m arena where the traverse spacing of two consecutive points was 0.5 m, and the longitudinal pitch was 0.4 m.

Note that the odor concentration distribution shown in Figure 10 is only one sample, but the plume distribution model should be built by many experiments. However, by analyzing several experimental data, it can be concluded that in the same experimental arena the odor distribution is similar whereas there exists some temperature or airflow difference in each experiment. That is, the gas concentration decreases downwind from the odor source, and increases crosswind from the plume border to the center. It can be seen in Figure 10 that, along the downwind direction, measurement values *R_s2_*/*R_02_* decrease from line 1 to line 6, that is, the gas concentration decreases from odor source to odor field edge. Along the crosswind direction, the gas concentration increases from odor field edge to center. This explains the fact that it is in accordance with the puff diffusion model of breeze condition described in Section 2.2. Except that gas concentration distribution is disturbed by the unsteady airflow, and odor plume is distorted to some extent to shape a curved feather. Since line 5 and line 6 are far away from the odor source, the concentration gradient is not sharp. This also proves that the concentration gradient of odor field is unstable [4]. Moreover, the olfaction robot was equipped with temperature sensor LM35, which could measure the ambient temperature in the room. To avoid determine *R_0_* (gas sensor resistance in air) before each experiment, the robot can find out it through the look-up table programmed on DSP to make temperature compensation.

**Figure 11.**
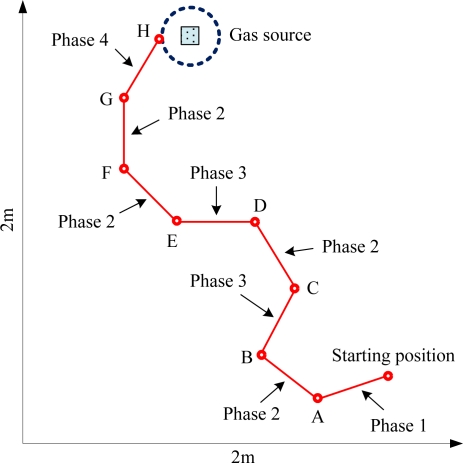
Typical trail of olfaction robot plume tracking.

According to mass experiments, some important parameters such as initial threshold, turning threshold, wind velocity threshold and robot steering velocity were properly determined. In order to validate the effectiveness of this search algorithm, several odor tracking experiments were conducted. During these experiments, both robot paths and search time were recorded for optimizing the parameters (thresholds, velocity, *etc.*) and improving the search efficiency. Experimental process was similar with gas concentration acquisition experiment whereas the robot was set to auto mode. Figure 11 shows a typical trail of olfaction robot plume tracking. During the search process, if olfactory robot moved to a new location, it stopped about 15 s to wait until all the gas sensor outputs were steady. Then it executed the plume tracing algorithm and sent data via WSN in 2 s. Besides, the robot spent 3 s to move to the next point. Thus the total search time was about 20 s and the movement distance of each step was 20–30 cm.

As the heading direction based algorithm described in Section 2.3.2, the whole plume tracking process was divided into four phases. For example, the olfactory robot entered phase 1 from the starting position in the low right corner of the arena. The gas concentration threshold *R_init* was set at 0.95. Then the robot could employ three gas sensors to check whether it was in the odor field. If outputs of the three sensors were below *R_init*, it indicated that the robot itself had been in the odor field, otherwise the robot continued to search for the odor field along the maximal gas concentration direction.

After phase 1, the robot began to track chemical plume upwind in phase 2. The wind velocity threshold *F_thresh* was set at 1, and the steering angle of the robot was 45° each time. At point A in Figure 11, since output of F_2_ (at the right side of the baffle) was above 1 while output of F_1_ was below 1, the robot turned 45° to the right and could exactly move towards to the odor source direction under the PID control algorithm. Since the wind could not blow to the beams of two wind sensors when the robot moved to point B where the hot-wire extended direction was parallel with the wind direction, the output of F_1_ had little difference from that of F_2_, and both of them were less than *F_thresh*, then the robot came to phase 3. The turning threshold *R_turning* was set at 0.75, and the turning angle was 60° in phase 3. Since the output of R_1_ was below R_3_ and *R_turning*, the robot turned 60° to the right and continued to track the odor source under the PID control algorithm. Accordingly, the robot gradually moved towards the odor source and converted its motion phases between phase 2 and phase 3 continually based on information fusion of gas sensors and airflow sensors. The robot started local search in phase 4, when it arrived at point G where the outputs of both F_1_ and F_2_ were above *F_thresh*. As the robot was close to the odor source in phase 4, the turning angle was only set at 30°. Particularly, since the output of R_1_ was less than R_2_, the robot turned 30° to the right at point G. If a contact pickup in the front of olfaction robot touched the edge of odor source, the robot would stop and enable the buzzer to alarm. So far the olfaction robot accomplished the odor tracking, as shown at point H in Figure 11.

**Figure 12.**
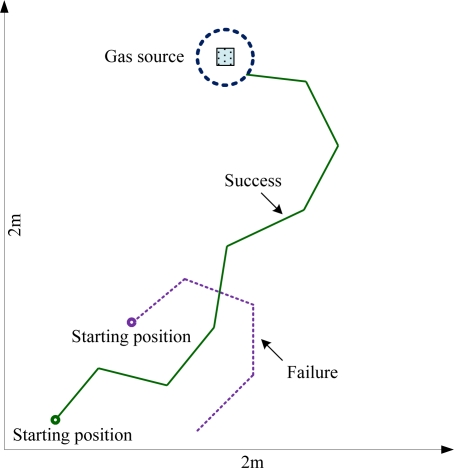
Odor source search path of olfaction robot.

Similar odor source search experiments under the same condition were repeated 10 times for evaluating the performance of olfactory robot, nine of the trials were successful. Figure 12 shows another successful trail (colored by green) at a different starting position. Also, the purple trail denotes a failed trial. The reason for this failure is that plume stabilization time was about 3–5 min after target odor was released in the closed room. For the sake of gas molecule diffusion, gas concentration tended to be uniform 20 min later. As the failed experiment was conducted after the odor field was used for a long time, the disappearance of the concentration gradient resulted in the plume tracking failure. Therefore, odor source search experiments should be controlled in 15 min. Experiments results show that the designed olfaction design performs well for the plume tracking task. Search time is less than 3 min and the number of search steps is less than 10. The search success ratio reaches 90% in a maximal search distance of 2 m.

### 4.3. Sound Source Search Trial

As shown in Figure 10, two hearing robots were randomly placed in the same experimental arena. When the olfactory robot found the odor source, it alarmed and sent a startup command via the WSN. Then two hearing robots acquired the sound signals from the buzzer in order to localize the sound, and moved towards the olfaction robot through the computed sound source direction angle. Since experiments were done in laboratory, the interference noises were mainly from echoes [44], motor noise and computer ventilator noise. Taking a hearing robot as an example, the detailed process of sound source search is described in the following.

In each search step, the hearing robot took 2 s to acquire audio signals and 5 s to execute sound localization as well as wireless data transmission. Besides, the robot spent 3 s to move to the next position. Thus the total search time was about 10 s and the movement distance was 20–30 cm at each step. As the sound localization and search algorithms described in Section 3.2, the whole search process is shown below. Firstly, the audio signals of four microphones were acquired at a sampling rate of 32 k and were filtered by FIR band-pass filters with band-pass frequency 200–8,000 Hz, so that low frequency and high frequency signals could be filtered. Then the audio signals were processed by Hanning windows and transformed by FFT. Secondly, short-time average zero-over rate was employed to detect the sound, which helped the robot to judge whether the current sound came from the buzzer or noise. Correspondingly, if it was noise, the robot would compute noise cross power spectrum (NCPS). Otherwise, the robot would compute sound cross power spectrum and got superimposition cross power spectrum by subtracting NCPS, so that interferences from environmental noise and circuits could be restrained. Thirdly, superimposition spectrum was processed by phase transformation to generate the weighted spectrum, and then transformed by IFFT to obtain the cross correlation function. By detecting the maximum of the cross correlation function, the time delay could be obtained. Finally, according to spherical interpolation introduced in Section 3.2.2, the hearing robot could obtain the sound source location and direction angle.

**Figure 13.**
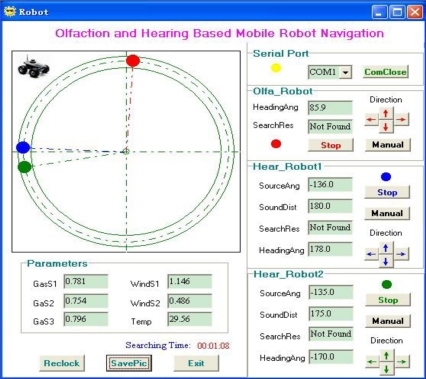
PC interface.

As mentioned above, the robot calculated the expected heading angle by adding its current heading angle and the computed sound source direction angle. Also, the robot could control its direction and velocity through PID algorithm and PWM duty cycle mapping. As shown in Figure 13, when a hearing robot moved forward with a heading angle of 178°, since the sound source direction angle calculated by sound localization procedure was −136°, the expected heading angle was 42°. Thus the robot could quickly approach the expected heading direction under PID control which had the advantages of small overshoot, high stability and precise control. After several steps, the hearing robot would stop when it was less than 20 cm (distance is computed by RSSI) from the olfactory robot. At this time the hearing robot found its companion and completed the search task.

In order to evaluate the performance of the hearing robot, similar experiments at the different starting positions of the same arena were conducted 10 times too, and all of them were entirely successful. Experimental results show that the angle range of sound localization is −180 + 180° with the accuracy of ±10°. In the maximal search distance of 2.5 m, the hearing robot can find the target sound source in 2 min using at most 10 steps. However, when the hearing robot is too close (<0.2 m) or far away (>3 m) from the sound source, the localization accuracy will decrease. Particularly, if there exist strong echoes or man-made noises, the robot may fail to find the sound source due to the deficiency of the algorithm itself [45]. Therefore, during the experiments, it should be as quiet as possible without man-made interference or other noise from ventilators. Additionally, since the accuracy of distance localization is low at the end of sound source search, and the ‘tentacles’ of the robot are long, robots may collide with each other so that sensors may be damaged. In order to avoid collision, a robot can judge the distance from the other robot via RSSI. When the distance is less than 20 cm, the robot will stop.

In summary, the olfactory robot can find the odor source target using at most 10 steps within the arena radius of 2 m, while two hearing robots can precisely find their companion namely the olfaction robot in 2 min (the above experiments were recorded in the video attachment).

## 5. Conclusions

This paper fuses smell, touch and hearing to design a multi-robot system. The multi-robot system, which integrates the techniques of gas sensor, airflow sensor, contact pickup, magnetoresistive sensor, microphone array, DSP and RF transceiver was successfully employed for plume tracking, sound localization and sound source search. Furthermore, robots in this system can communicate with each other and sink node via a WSN.

The steady-state diffusion model of continuous point source under the breeze condition is built by the integral model of mobile puff diffusion which is different from the Gaussian plume model where airflow velocity is large and the downwind diffusion is ignored. Also, the built puff diffusion model is verified by the real plume distribution of the experimental environment. On this basis, a multi-sensor information fusion step-by-step search algorithm is proposed. The olfactory robot can determine the gas source direction angle according to the wind direction and the gas concentration gradient calculated by fusing the output information of three gas sensors and two airflow sensors. Compared with the traditional plume edge search method, since the crosswind plume is relatively broad in the breeze condition, the olfactory robot rarely leaves the plume before the odor source is found.

A heading direction based robot motion control method is presented. The robot can accurately adjust its heading direction according to the deviance between the current heading direction measured by magnetoresistive sensor and the expected heading direction acquired by odor/sound localization algorithms. Compared to the traditional open-loop motion control method, the close-loop PID control algorithm can continuously and steadily control the movement velocity and direction of the robot so that it improves the search efficiency and success ratio. Experimental results show that the olfactory robot can find the odor source in the distance of 2 m with a 90% success ratio, while the hearing robots can achieve the 360° omnidirectional localization, and the sound localization error is less than ±10°. The devised multi-robot system performs quite well for the target search.

Work is under way to deep study the biological inspiration, strengthen the integration of the two sensory modalities, exploit the fusion of more biological senses such as vision and touch in combination with the olfaction and to further the performance of the multi-robot system. For example, video sensor and ultrasonic sensor can help robots to achieve target recognition, range acquisition and obstacle avoidance. Particularly, most of the current plume tracking experiments are conducted in the stable indoor wind field. The selective search for multiple odor sources and robot navigation in the outside natural environment will be studied in the future.

## Figures and Tables

**Table 1.** PID control algorithm.

*γ = β* − *α* //Heading angle deviation *γ*
if (*γ* > 180.0) //Optimize the direction
*γ = γ* − 360
if (*γ* < −180.0)
*γ= γ +* 360
P_value_ = K_P_ × *γ* //Proportion value
if (|*γ*|< 20.0)
I_value_ = I_value_ + K_I_ × *γ* × Timelnterval //Integral value
else
I_value_ = 0.0
if (I_value_ >30.0) //Set the threshold of I_value_
I_value_ = 30.0
else if (I_value_ < −30.0)
I_value_ = −30.0
D_value_ = K_D_ × (*γ* − previous *γ*) / TimeInterval //Differential value
if (D_value_ > 20.0) //Set the threshold of D_value_
D_value_ = 20.0
else if (D_value_ < −20.0)
D_value_ = −20.0
previous *γ = γ*
*ɛ* = P_value_ + I_value_ + D_value_ //error output *ɛ*

**Table 2.** Duty cycle mapping.

if ((*ɛ* > L) || (*ɛ* < − L))
{
DutyRate_Left = 0.01×*ɛ*
DutyRate_Right = −0.01×*ɛ*
}
if (*ɛ* ≥ 0)
{
DutyRate_Left = (0.01 −1) / L × *ɛ* + 1
DutyRate_Right = −(0.01 +1/ L) × *ɛ* + 1
}
else
{
DutyRate_Left = (0.01 + 1) / L × *ɛ* + 1
DutyRate_Right = (−0.01 + 1/ L) × *ɛ* + 1
}

**Table 3.** Motion models of mobile robots.

**Number**	**Motion modes**	**Left motor**	**Right motor**
1	Fast left turn	Backward	Forward
2	Fast right turn	Forward	Backward
3	Slow left turn	Slow down	Speed up
4	Slow right turn	Speed up	Slow down
5	Go forward	Forward	Forward

**Table 4.** Wireless data communication protocol.

0	1	2	3	4	5	6	7	8
FA	FB	Addr	Fn	Bit1	Bit2	Bit3	Bit4	check

**Table 5.** Function definition of Fn*.

**Fn**	**Functional description**	**Fn**	**Functional description**
0	Stop	9	Airflow sensor 1
1	Run	10	Airflow sensor 2
2	Set heading angle	11	Temperature
3	Auto mode	12	Odor source search result
4	Manual mode	13	Sound source direction angle
5	Current heading angle	14	Distance from sound source
6	Gas sensor 1	15	Distance from olfactory robot
7	Gas sensor 2	16	Sound source search result
8	Gas sensor 3	-	-

* Notes: (1) Bit4 is the decimal bit for the heading angle, while for other data, Bit3 and Bit4 are the decimal bits. (2) Search result: not found-0 and found-1. (3) Unused data bytes are assigned as 0 × FF or 0 × 00. (4) Communication bandwidth of WSN < 128 K. The wireless network realizes data transfer between different nodes based on the above communication protocol. Particularly, this protocol makes it possible that new extended information can be added easily.
